# Correction to “The Clinicopathological Characteristics of Muscle‐Invasive Bladder Recurrence in Upper Tract Urothelial Carcinoma”

**DOI:** 10.1111/cas.70101

**Published:** 2025-05-14

Shigeta K, Matsumoto K, Ogihara K, et al. The clinicopathological characteristics of muscle‐invasive bladder recurrence in upper tract urothelial carcinoma. *Cancer Sci*. 2021;112:1084‐1094. https://doi.org/10.1111/cas.14782

Figures 3A and 3B in the above article are incorrect. The correct images are shown below:

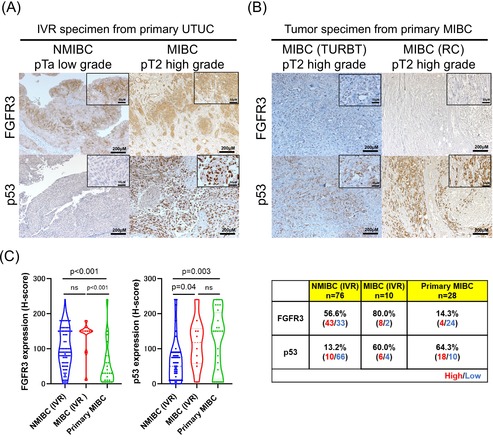

We apologize for this error.

